# *Rhizoma Coptidis* Inhibits LPS-Induced MCP-1/CCL2 Production in Murine Macrophages via an AP-1 and NF*κ*B-Dependent Pathway

**DOI:** 10.1155/2010/194896

**Published:** 2010-06-21

**Authors:** Andrew Remppis, Florian Bea, Henry Johannes Greten, Annette Buttler, Hongjie Wang, Qianxing Zhou, Michael R. Preusch, Ronny Enk, Robert Ehehalt, Hugo Katus, Erwin Blessing

**Affiliations:** ^1^Medizinische Klinik III, Universität Heidelberg, Im Neuenheimer Feld 410, 69120 Heidelberg, Germany; ^2^Deutsche Gesellschaft für Traditionelle Chinesische Medizin (DGTCM), 69126 Heidelberg, Germany; ^3^Institute of Biomedical Sciences Abel Salazar, Porto University, 4099-002 Porto, Portugal; ^4^Department of Environmental and Occupational Health Sciences, University of Washington, Seattle, WA 98195-7234, USA; ^5^Medizinische Klinik IV, Universität Heidelberg, 69120 Heidlberg, Germany

## Abstract

*Introduction*. The Chinese extract *Rhizoma coptidis* is well known for its anti-inflammatory, antioxidative, antiviral, and antimicrobial activity. The exact mechanisms of action are not fully understood. *Methods*. We examined the effect of the extract and its main compound, berberine, on LPS-induced inflammatory activity in a murine macrophage cell line. RAW 264.7 cells were stimulated with LPS and incubated with either *Rhizoma coptidis* extract or berberine. Activation of AP-1 and NF*κ*B was analyzed in nuclear extracts, secretion of MCP-1/CCL2 was measured in supernatants. *Results*. Incubation with *Rhizoma coptidis* and berberine strongly inhibited LPS-induced monocyte chemoattractant protein (MCP)-1 production in RAW cells. Activation of the transcription factors AP-1 and NF*κ*B was inhibited by *Rhizoma coptidis* in a dose- and time-dependent fashion. *Conclusions. Rhizoma coptidis* extract inhibits LPS-induced MCP-1/CCL2 production *in vitro* via an AP-1 and NF*κ*B-dependent pathway. Anti-inflammatory action of the extract is mediated mainly by its alkaloid compound berberine.

## 1. Introduction

*Rhizoma coptidis* is a commonly used herb in Chinese medicine. It shows anti-inflammatory, anti-oxidative, anti-viral, and anti-microbial activity and is therefore used for a number of different medical conditions, mainly for dermatological disorders including acne, neurodermatitis and skin ulcers. The main compound of *Rhizoma coptidis*, the benzylisoquinoline alkaloid berberine, was also shown to display beneficial effects on conditions associated with hyperglycemia [1–4] and to lower serum cholesterol [5]. The potent actions of *Rhizoma coptidis* and berberine have been investigated in a number of different cell lines, such as keratinocytes [6], cancer cells [7–9], human hepatoma cells [10], vascular smooth muscle cells [11, 12], and HepG2 cells [13]. However there is only limited mechanistic data on the effects of *Rhizoma coptidis* and berberine and they are mainly limited to *in vitro* studies. Few studies have investigated *Rhizoma coptidis* or berberine in animal models. Total alkaloids from *Rhizoma coptidis* proved to be protective against *H. pylori* LPS-induced gastric lesions in rats [14]. In another study, a combination of herbal extracts, including components of *Rhizoma coptidis* showed anti-inflammatory activities as potent as the effects observed with high doses of celecoxib or dexamethasone in acute and chronic inflammation models [15]. 

Despite the well-described anti-inflammatory action, there is little data on interactions of the total extract or berberine on mononuclear cells. The transcription factor activator protein 1 (AP-1) plays a critical role in inflammation and carcinogenesis. Nuclear factor-kappaB (NF*κ*B) is involved in the regulation of cytokine production. Monocyte chemoattractant protein 1 (MCP-1/CCL2) is a cytokine that attracts blood monocytes and tissue macrophages and is therefore involved in chronic inflammatory disorders, for example, atherosclerosis.

In the present paper, we examined the effect of the extract and its main compound, berberine, on LPS-induced inflammatory activity in RAW 264.7 cells, a mouse leukaemic macrophage cell line.

## 2. Methods

### 2.1. Preparation of Rhizoma Coptidis Extract and Berberine

Ten gramm *Rhizoma coptidis* were washed with distilled water, dried and cut into small pieces. Herbs were diluted in 100 ml water and boiled for 2 hours. The solute was percolated through filter paper (Whatman, pleated filter grade 597 1/2, 4–7 *μ*m) and then sterilized by filtration through a 0.2 *μ*m pore filter (Minisart-plus, Sartorius). Resulting *Rhizoma coptidis* extract was stored in aliquots at −20°C until use. 

Berberine, one of the main active alkaloids of *Rhizoma coptidis*, was purchased from Sigma (Taufkirchen, Germany) The substrate was diluted in methanol to generate a stock solution with a final concentration of 10^−2^ mol. For the experiments, the stock solution was diluted with serum free medium (final total volume 4 mL) to generate concentrations of 10^−3^, 10^−4^, 10^−5^, and 10^−6^ mol for the cell culture experiments.

### 2.2. Cell Culture

The murine macrophage cells (RAW 264.7; ATCC, Manassas, VA) were grown in RPMI (PAA, Pasching, Austria) and supplemented with 10% heat-inactivated FCS. Cells were seeded at a density of 2.5 × 10^6^ per 10 cm culture dish. After attachment and before start of the experiments, cells were changed to serum-free medium (RPMI). *Rhizoma coptidis* or berberine was added in various dilutions for different time points. LPS (Sigma, Taufkirchen, Germany) was diluted in serum-free medium in a final concentration of 2 *μ*g/mL and added to the cells 30 minutes prior end of the incubation period. To compare anti-inflammatory activity, effects of several statins (10^−4^ mol rosuvastatin; 10^−4^ mol fluvastatin) and angiotensin receptor blockers (10^−4^ mol olmesartan, 10^−4^ mol telmisartan) were also evaluated (exposure time 240 min) on LPS-stimulated RAW 264.7 cells.

### 2.3. Preparation of Nuclear Extracts

Nuclear extracts were isolated using the method of Hoppe-Seyler et al. [16]. Briefly, cells were washed 3x with PBS and lysed directly on the culture dish in 1.0 mL cold RNA lysis buffer (0.6% NP40, 0.15 M NaCl, 10 mM Tris pH 7.9 and 1 mM EDTA) and the nuclear proteins were extracted into 50 *μ*l cold extraction buffer containing 10 mM Hepes pH 7.9, 0.1 mM EGTA, 0.1 mM EDTA, 1.5 mM MgCl_2_, 420 mM NaCl, 25% glycerol and a proteinase inhibitor cocktail containing AEBSF, pepstatin A, E-64, bestatin, leupeptin and aprotinin (Sigma, Taufkirchen, Germany) and stored at −80°C. Protein was measured using the Bradford protein dye reagent (Bio-Rad, Munich, Germany).

### 2.4. Transcription Factor Activity

For electrophoretic mobility shift assays (EMSA), a double-stranded oligonucleotide (Santa Cruz) representing the consensus-binding site for AP-1 and NF*κ*B were radiolabeled with *γ*-^32^P-ATP using T-4 polynucleotide kinase (Promega, Madison, WI). The labeled oligonucleotides were incubated with 5 *μ*g of nuclear proteins and loaded on a 4% nondenaturating acrylamide gel for separation from the unbound oligonucleotides according to the manufacturer's manual (Promega). To demonstrate the specificity of the EMSA, cold competition was tested in each individual assay. Gels were analyzed by phosphorimaging (Cyclone, Packard Instruments, Meriden, CT).

### 2.5. Inflammatory Mediators in Supernatants

Supernatants from LPS-stimulated cells, with and without incubation with either *Rhizoma coptidis*, berberine (10^−4^ mol), statins (10^−6^ mol rosuvastatin, 10^−6^ mol fluvastatin), or angiotensin receptor blockers (10^−5^ mol telmisartan, 10^−5^ mol olmesartan) were obtained and stored at −80°C until use. MCP-1/CCL2, interleukin-1 (IL-1)-beta, and interleukin-12 (IL-12) concentrations were measured with a mouse Elisa kit (R&D Systems, Wiesbaden, Germany) following the manufacturer's protocol.

Nitric oxide (NO) is a gaseous free radical with a short half-life of a few seconds or less. Therefore, the levels of the more stable NO metabolites, nitrite (NO_2_^−^) and nitrate (NO_3_^−^), have been used in the indirect measurement of NO in biological fluids. Nitrate was converted to nitrite using nitrate reductase. Total nitrite and endogenous nitrite were measured in supernatants using a colorimetric assay (R&D Systems, Wiesbaden, Germany). To obtain the nitrate concentration, endogenous nitrite was subtracted from the total nitrite value.

### 2.6. Statistical Analysis

Statistical analysis was performed using the unpaired Students *t*-test. Data are presented as mean ± S.E.M., and values of *P* < .05 were considered statistically significant. All experiments were performed at least three times and representative results are shown.

## 3. Results

### 3.1. Morphology

**Figure 1:**
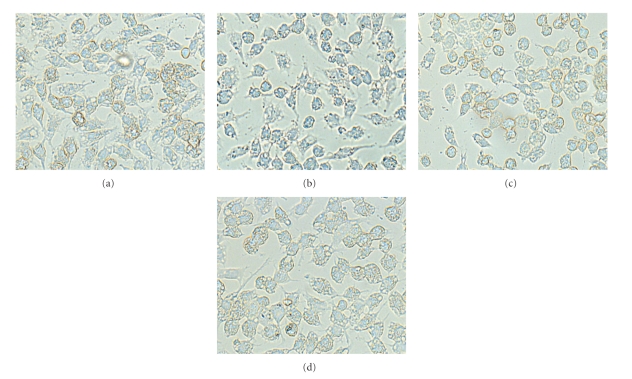
Morphology of RAW 264.7 cells cultured in standard medium (a), after stimulation with LPS (b), after LPS-stimulation and exposure to either total extract of *Rhizoma coptidis* (dilution 1 : 5) (c), or to berberine (10^−4^ mol) (d). Activation with LPS resulted in moderate reduction of total cell numbers and moderate altered cell morphology. No apparent morphologic changes were observed between the different LPS-stimulated cells.

Morphology and total protein count of adherent RAW 264.7 cells did not differ between LPS-stimulated control cells and LPS-stimulated cells, preincubated with various dilutions of *Rhizoma coptidis *extract (between 1 : 2 and 1 :  20), even after exposure times of up to 360 min (dilution 1 : 5), or with berberine in concentrations of up to 10^−3^ mol. Representative cell morphology is displayed on Figure 1.

LDH concentrations were slightly higher in supernatants from LPS-stimulated cells (31.3 ± 0.6 U/l, *P* < .05) as compared with control cells. No significant differences were found in supernatants from LPS-stimulated cells exposed to *Rhizoma coptidis *(24.3 ± 14.6 U/l), and from LPS-stimulated cells exposed to berberine (30.3 ± 18.7 U/l), as compared to supernatants from control cells (17.6 ± 6.7 U/l); (supernatants from the highest concentrations of *Rhizoma coptidis* and berberine evaluated; data are mean from 3 independent experiments). No significant differences were observed between the different LPS-stimulated cells.

### 3.2. AP-1 Activity

**Figure 2:**
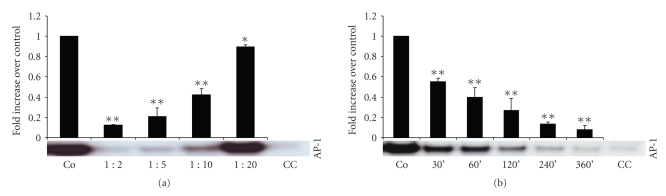
Binding activity of the transcripton factor AP-1 in nuclear extracts of LPS-stimulated RAW 264.7 cells. Effects of total extract of *Rhizoma coptidis* was evaluated with different concentrations (incubation time 240 min), (a) and exposure times (dilution 1 : 5), (b) and was compared with transcription factor activation in LPS-stimulated control cells. Values represent results from at least three independent experiments. AP-1: activated protein-1, Co: LPS-stimulated control cells, and CC: cold competition. *P < .005, **P < .001.

Incubation of LPS-stimulated RAW cells with *Rhizoma coptidis* inhibited AP-1 activity in a concentration (incubation time 240 minutes), (Figure 2(a)) and time dependent fashion (dilution 1 : 5), (Figure 2(b)). For example, AP-1 activity in nuclear extracts of LPS-stimulated RAW 264.7 cells was reduced by more than 90% after preincubation for 360 min with 1 : 5 diluted *Rhizoma coptidis* extract, as compared to nuclear extracts from LPS-stimulated control cells. Significant reduction of LPS-induced activation of AP-1 was already observed as early as after 30 min of preincubation (dilution 1 : 5), (Figure 2(b)). Profound reduction of AP-1 activity was still observed 48 hours postexposure to *Rhizoma coptidis* (data not shown).

### 3.3. NF*κ* B Activity

**Figure 3:**
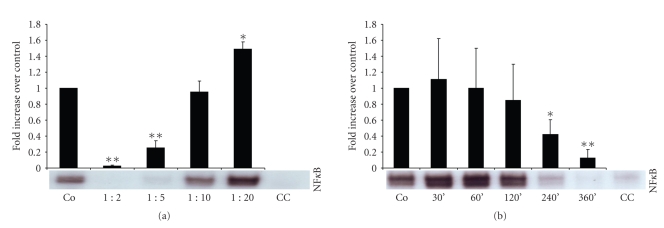
Gel-shift analysis of the transcripton factor NF*κ* B. Treatment with *Rhizoma coptidis* inhibited binding activity in a dose-dependent (incubation time 240 min), (a) and time-dependent (dilution 1 : 5), (b) fashion. NF*κ* B: nuclear factor*κ* B, Co: LPS-stimulated control cells, and CC: cold competition. *P < .005, **P < .001.

Significant reduction of transcription factor NF*κ* B activity required higher concentrations (Figure 3(a)) and longer preincubation times (Figure 3(b)) with *Rhizoma coptidis* extract, as compared with effects on AP-1 activity. Statistical significant reduction was observed with dilutions of up to 1 : 5 (incubation time 240 min), (Figure 3(a)) and after incubation times of 240 min or longer (dilution 1 : 5), (Figure 3(b)). Surprisingly, low concentrations of *Rhizoma coptidis* extract (1 : 20, incubation time 240 min) caused a mild but significant increase of NF*κ* B activity (*P* < .005), (Figure 3(a)). Profound downregulation of NF*κ* B activity at higher concentrations (1 : 2 and 1 : 5) persisted 48 hours after exposure to the total extract. Again, exposure to low concentrations (1 : 20) of *Rhizoma coptidis* resulted in enhanced NF*κ* B activity (data not shown).

### 3.4. Effects of Berberine

**Figure 4:**
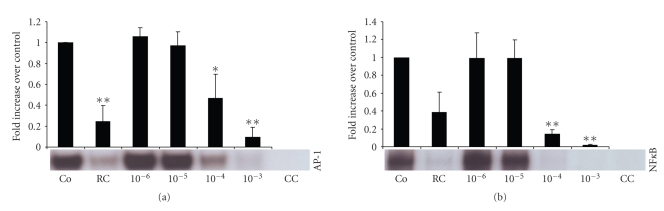
The main alkaloid compound of *Rhizoma coptidis*, berberine, inhibited binding activity of AP-1 (a) and NF*κ* B (b) in a concentration dependent fashion. Inhibitory potential of berberine was directly compared with the total extract of *Rhizoma coptidis* (dilution 1 : 5, incubation time 240 min). LPS-stimulated cells were incubated for 240 min. Berberine concentrations listed represent mol. AP-1: activated protein-1, NF*κ* B: nuclear factor*κ*B, Co: LPS-stimulated control cells, *RC:Rhizoma coptidis*, Be: berberine, and CC: cold competition. *P < .05, **P < .001.

Incubation with the main alkaloid compound of *Rhizoma coptidis*, berberine, also significantly inhibited binding activity of AP-1 (Figure 4(a)) and NF*κ* B (Figure 4(b)), at concentrations of 10^−4^ mol or higher (incubation time 240 min). The pattern and magnitude of the inhibitory effects of berberine on transcription factor activity suggests that it represents the main anti-inflammatory compound of *Rhizoma *coptidis.

### 3.5. Comparison with other Anti-Inflammatory Agents

Incubation with the main alkaloid compound of *Rhizoma coptidis*, berberine, also significantly inhibited binding activity of AP-1 (Figure 4(a)) and NF*κ* B (Figure 4(b)), at concentrations of 10^−4^ mol or higher (incubation time 240 min). The pattern and magnitude of the inhibitory effects of berberine on transcription factor activity suggests that it represents the main anti-inflammatory compound of *Rhizoma *coptidis.

**Figure 5:**
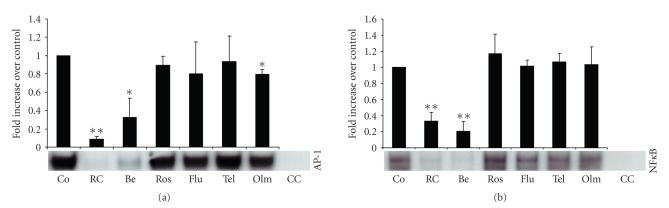
Direct comparison of cholesterol synthase inhibitors, angiotensin receptor blockers, total extract of *Rhizoma coptidis *(dilution 1 : 5), and berberine (dilution 10^−4^ mol) on transcription factor activity of AP-1 (a) and NF*κ* B (b). Exposure time of all substrates was 240 min. AP-1: activated protein-1, NF*κ* B: nuclear factor*κ*B, Co: LPS-stimulated control cells, *RC:Rhizoma coptidis*, Be: berberine, Ros: 10^−6^ mol rosuvastatin, Flu: 10^−6^ mol fluvastatin, Tel: 10^−5^ mol telmisartan, Olm: 10^−5^ mol olmesartan, and CC: cold competition. *P < .005, **P < .001.

HMG-Co enzyme A reductase inhibitors (statins) as well as ACE-inhibitors and angiotensin receptor blockers (ARBs) are known for their potent anti-inflammatory effects. Preincubation with the ARB olmesartan resulted in a small, but significant reduction of AP-1 activity in LPS-stimulated RAW cells (*P* < .005); however, the effect was by far less profound as compared with *Rhizoma coptidis* extract (dilution 1 : 5) and with berberine (10^−4^ mol), (Figure 5(a)). All other tested statins and ARBs failed to show significant effects on AP-1 activity, (Figure 5(a)). No significant effects on NF*κ* B activity were observed with any of the tested statins and ARBs, (Figure 5(b)).

### 3.6. Secretion of MCP-1/CCL2, IL-1 beta, IL-12, and NO

**Figure 6:**
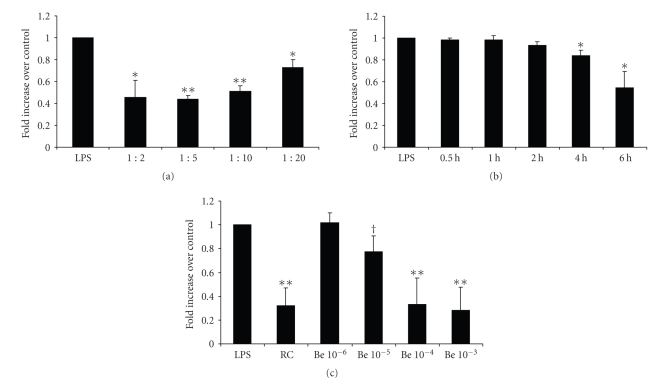
Secrection of MCP-1/CCL2 was evaluated with ELISAs of supernatants. Supernatants were collected after preincubation with different concentrations (a) and exposure times (b) of *Rhizoma coptidis. *Berberine also inhibited MCP-1/CCL2 secretion in a concentration-dependent fashion (c). Berberine concentrations listed represent mol. LPS: lipopolysaccharide, *RC:Rhizoma coptidis*, Be: berberine, MCP-1/CCL2: monocyte chemoattractant protein-1. *P < .005, **P < .001. AP-1: activated protein-1, Co: LPS-stimulated control cells, and CC: cold competition. ^†^ *P* < .05, *P < .005, **P < .001.

Secretion of monocyte chemoattractant protein-1 by LPS-stimulated RAW 264.7 cells was also significantly reduced in a concentration- (Figure 6(a)) and time-dependent (Figure 6(b)) fashion, as evaluated by ELISAs of supernatants. Following the results of the *Rhizoma coptidis *assays, higher concentrations of berberine potently inhibited MCP-1/CCL2 secretion (Figure 6(c)). Again, preincubation of stimulated cells with various statins and ARBs did not inhibit LPS-induced secretion of MCP-1/CCL2 by RAW cells (data not shown). In contrast to the results of the transcription factor essays, MCP-1/CCL2 secretion was significantly inhibited already after pretreatment with low concentrations of* Rhizoma coptidis *or berberine (Figure 6(c)), suggesting that inhibitory effects on cytokine secretion might not exclusively be mediated via AP-1 and NF*κ* B. MCP-1/CCL2 levels were suppressed by *Rhizoma coptidis* (218 pg/mL and 956 pg/mL) but not by berberine (2125 pg/mL and 2269 pg/mL) as compared with LPS-stimulated control cells (2335 pg/mL and 2355 pg/mL), 24 hours, respectively, 48 hours post exposure.

Surprisingly, concentration of interleukin-1 beta and interleukin-12, two inflammatory mediators, was below detectable levels in all supernatants tested, using standard ELISA kits (R&D Systems, Wiesbaden, Germany) (data not shown). It is possible that cell numbers in the present setting and therefore cytokine secretion was too low to reach detectable levels of IL-1 beta and IL-12. Production of total nitrite (NO_2_^−^) was significantly inhibited by *Rhizoma coptidis *(245 ± 25.7 pg/mL, dilution 1 : 2, *P* < .001; 329 ± 28.8 pg/mL, dilution 1 : 5, *P* < .001; 407 ± 27.6 pg/mL, dilution 1 : 10, *P* < .001; 512 ± 44.7 pg/mL, dilution 1 : 20, *P* < .05), as compared to LPS-stimulated RAW cells (635 ± 16.9 pg/mL). Incubation with berberine in a concentration of 10^−4^ mol did not inhibit production of total nitrite (651 ± 32.5 pg/mL, n.s.). Endogenous nitrite was below detectable levels in all supernatants evaluated; therefore, nitrate concentration equals total nitrite concentration in our assays.

## 4. Discussion

The effect of *Rhizoma coptidis* and its possible mechanisms have been evaluated in a number of different cell lines. Since *Rhizoma coptidis* is well established in the treatment of common dermatological disorders, Enk et al. investigated the effect of the extract on TNF-*α* induced NF*κ*B translocation in human keratinocytes [6]. Translocation of NF*κ*B into the cell nucleus after stimulation with TNF-*α* could be inhibited in a dose-dependent fashion by the total extract, but not by its main alkaloid compound berberine. Authors conclude that berberine exerts its anti-inflammatory effects by inhibiting signal transduction pathways other than the NF*κ*B dependent pathway. In the present study, MCP-1/CCL2 secretion of RAW cells was significantly inhibited already after incubation with concentrations of* Rhizoma coptidis *or berberine that did not significantly inhibit transcription factor activation, suggesting that inhibitory effects on cytokine secretion might not exclusively be mediated via AP-1 and NF*κ*B. The pattern and magnitude of the inhibitory effects of berberine on transcription factor activity in our experiments supports the hypothesis, that it represents the main anti-inflammatory compound of *Rhizoma coptidis.*

Berberine displays a number of potential beneficial effects in cancer cells and might therefore also exerts anticancer properties [8, 9, 17]. The alkaloid induces production of reactive oxygen species (ROS) and downregulation of several matrix metalloproteinases (MMP-1) both on mRNA as well as on protein level [7]. Another study showed that berberine suppresses invasion of cancer cells through different signalling pathways resulting in inhibition of MMP-2 [8] and MMP-9 [8, 9]. MMPs not only play a role in carcinogenesis; matrix-degrading proteases can destabilize atherosclerotic lesions and therefore also play an important role in advanced cardiovascular disease. Oxidative modification of low-density lipoprotein (LDL) is a crucial step in rather earlier stages of atherogenesis. Again, in a study by Hsieh et al., berberine was shown to inhibit generation of ROS but also to reduce LDL oxidation and to prevent oxLDL-induced cellular dysfunction in endothelial cells [18].

Our study is one of few reports of the anti-inflammatory activity of *Rhizoma coptidis* in a macrophage cell line [19–22]. This is of particular interest, since macrophages play a crucial role in various stages of atherosclerosis, and its clinical sequelae coronary artery disease and stroke. Atherosclerotic lesions develop as a result of a sustained immune response to chronic inflammatory processes in the vessel wall, often caused by endothelial injury [23]. Monocytes play a crucial role in initiating and maintaining vascular inflammation. Monocytes convert to macrophages, consume oxidized lipids, and subsequently form characteristic foam cells. Foam cells then again secrete proinflammatory cytokines which perpetuate the inflammatory response, leading eventually to fatty streak formation. After fatty streaks are established, macrophages constitutively secrete proatherosclerotic mediators, for example, inflammatory proteins, MMPs, adhesion molecules, and chemokines such as MCP-1/CCL2 [24]. MCP-1/CCL2 further attracts circulating monocytes and tissue macrophages and therefore contributes to the sustained inflammation within the vessel wall. 

Our observations, that *Rhizoma coptidis* exerts its anti-inflammatory mechanisms, at least in part, through inhibitory effects on MCP-1/CCL2 production, is supported by studies of Ko et al.. Zoagumhwan water extract, a Korean herbal remedy as well as berberine inhibited angiotensin II-induced MCP-1/CCL2 expression and monocyte adhesion to human umbilical vein endothelial cells [25]. In the same line, berberine was shown to inhibit the expression of TNFalpha, IL-6, and MCP-1/CCL2 in acLDL-stimulated macrophages [26]. In contrast to our observations, Li et al. report of potential proatherosclerotic effects of berberine. The compound induced foam cell formation in RAW 264.7 cells as well as in mouse and human primary macrophages by upregulating scavenger receptor A expression [27]. Authors conclude that promotion of foam cell formation, a hallmark in early atherogenesis, could therefore counter-balance the beneficial effect of berberine on serum cholesterol levels, which is believed to be mediated by inducing LDL receptors in hepatic cells [5, 28]. We strongly believe that the well-described potent anti-inflammatory actions of berberine by far outweigh its potential effects on stimulating uptake of modified LDL *in vitro*. In the present paper, we focused on anti-inflammatory mechanisms of both *Rhizoma coptidis* and berberine on a transcriptional and post-transcriptional level. Assessment of foam cell formation or even induction of early atherosclerotic lesions was not subject of the present study.

Few studies investigated the effects of *Rhizoma coptidis* or berberine in *in vivo* models. The total extract was shown to be protective against *H. pylori* LPS-induced gastric mucosal inflammation. The concerned mechanisms seem to be related to its inhibition on epithelial cell apoptosis, upregulating cNOS, and reducing serum concentrations of TNF-alpha [14]. The observation that Rhizoma coptidis acts through TNF-alpha inhibition is consistent with results of our study, since NF*κ*B and AP-1 are the two main downstream effectors of TNF-alpha. Berberine was also shown to display beneficial effect on conditions associated with hyperglycemia [1–4]. The alkaloid prevents fructose-induced insulin resistance in rats by promoting hepatocyte nuclear factor-4alpha [1]. Berberine also increases insulin expression and antioxidant enzyme activity, promotes beta cell regeneration, and decreases lipid peroxidation in diabetic rats [4].

Park et al. used a croton oil-induced ear edema model and an acetic acid-induced capillary permeability test to evaluate the effects of a combined herbal preparation (RAH13), of which *Coptis chinensis* is a main compound. Models of chronic inflammation were also tested, using the cotton pellet test and a delayed-type hypersensitivity test. Oral administration of RAH13 showed anti-inflammatory activity *in vivo* as potent as with high doses of celecoxib or dexamethasone [15].

Several substances and drugs, mainly statins and ACE-inhibitors or ARBs are thought to improve outcome in cardiovascular patients, at least in part, via their anti-inflammatory action. In the present study, anti-inflammatory activity of *Rhizoma coptidis* exceeded those observed with various statins and ARBs. With its potent inhibitory effects on transcription factor activity and MCP-1/CCL2 secretion* in vitro*, together with its well described lipid lowering and hypoglycemic properties, *Rhizoma coptidis* might be of potential benefit for patients with atherosclerotic disease. Further *in vivo* and clinical studies seem to be warranted to elucidate the potential role of *Rhizoma coptidis* in the prevention or treatment of patients with metabolic syndrome or cardiovascular disease.
